# Healthcare Cost Savings Associated with Increased Whole Grain Consumption among Australian Adults

**DOI:** 10.3390/nu13061855

**Published:** 2021-05-29

**Authors:** Mohammad M. H. Abdullah, Jaimee Hughes, Sara Grafenauer

**Affiliations:** 1 Department of Food Science and Nutrition, Kuwait University, Kuwait City 10002, Kuwait; 2 Grains & Legumes Nutrition Council, 1 Rivett Rd, North Ryde 2113, Australia; j.hughes@glnc.org.au (J.H.); sarag@glnc.org.au (S.G.); 3 School of Medicine, University of Wollongong, Northfields Avenue, Wollongong 2522, Australia

**Keywords:** whole grains, cardiovascular disease, diabetes, healthcare cost, cost saving analysis, nutrition economics

## Abstract

Many dietary guidelines emphasise “mostly” whole grain food choices as part of an overall healthy eating pattern based on evidence for enhancing nutritional status and reducing chronic disease. Still, countries including Australia fall short of their consumption targets. Furthermore, healthcare cost savings associated with increasing the consumption of whole grains in alignment with the Daily Target Intake (DTI) recommendation of 48 g are unknown. The aim of this study was to assess the potential savings in costs of healthcare and lost productivity associated with a reduction in the incidence of Type 2 Diabetes Mellitus (T2DM) and cardiovascular disease (CVD) through meeting the 48 g DTI recommendation for whole grains among the Australian adult population (>20 years). A three-step cost-of-illness analysis was conducted using input parameters from: 1) estimates of proportions of consumers (5%, 15%, 50%, and 100%) who would increase their current intake of whole grains to meet the recommended DTI in Australia; 2) relative reductions in risk of T2DM and CVD associated with specific whole grain consumption, as reported in meta-analysis studies; and 3) data on costs of healthcare and productivity loss based on monetary figures by national healthcare authorities. A very pessimistic (5% of the population) through to universal (100% of the population) adoption of the recommended DTI was shown to potentially yield AUD 37.5 (95% CI 22.3–49.3) to AUD 750.7 (95% CI 445.7–985.2) million, and AUD 35.9 (95% CI 8.3–60.7) to AUD 717.4 (95% CI 165.5–1214.1) million in savings on annual healthcare and lost productivity costs for T2DM and CVD, respectively. Given such economic benefits of the recommended consumption of whole grains, in exchange for refined grains, there is a real opportunity to facilitate relevant socioeconomic cost-savings for Australia and reductions in disease. These results are suggestive of a much greater opportunity to communicate the need for dietary change at all levels, but particularly through food-based dietary guidelines and front-of-pack labelling initiatives.

## 1. Introduction

Grains and grain-based foods are a key food category in dietary recommendations, as they provide 60% of global energy intake [1] with a range of important nutrients, including dietary fibre, folate, thiamin, magnesium, and iron [2]. Since 1979, the Australian Dietary Guidelines have promoted whole grain choices [3], which have been described by Food Standards Australia New Zealand (FSANZ) [4] as the intact grain or the dehulled, ground, milled, cracked or flaked grain where the constituents—endosperm, germ and bran—are present in such proportions that represent the typical ratio of those fractions occurring in the whole cereal, and includes wholemeal. The current dietary guidelines point to “mostly” whole grain and/or high cereal fibre varieties, and provide examples such as breads, cereals, rice, pasta, noodles, polenta, couscous, oats, quinoa, and barley [5], while recommending that two-thirds of the total daily grain intake are whole grain. In Australia, wholemeal bread is the leading source of whole grain, followed by ready-to-eat whole grain breakfast cereal [6].

Whole grain consumption and promotion is a key focus of healthy dietary recommendations worldwide. Research ranks diets low in whole grain as second only to diets high in sodium in causing greatest risks of morbidity and mortality [7]. This is also the case in Australia, where 27,500 preventable deaths are due to diet, with 7400 due to diets low in whole grain annually [8]. Although whole grain foods are promoted through dietary guidelines and other health promotion organisations, consumption in Australia remains low. The Daily Target Intake (DTI) is 48 g for Australians ≥ 9 years of age [9]. However, the most recent National Nutrition and Physical Activity Survey (NNPAS), based on single 24 h dietary recall data (*n* = 12,153) [6], demonstrated a median daily whole grain intake of 21 g in adults (19–85 years), indicating a mean gap of 27 g/day between current and targeted consumption. In contrast, 70% of grains consumed in Australia are refined grains, such that a 160% increase in current whole grain consumption and a 30% decrease in refined grain (cereal) food consumption has been recommended by the Australian Dietary Guidelines [10].

The Australian Institute of Health and Welfare (AIHW) estimated that annual health expenditure (direct costs) in 2015/16 from cardiovascular disease (CVD) was the second highest of all disease groups in the country, at approximately AUD 10.5 billion, representing approximately 10% of total health expenditure [11]. Additionally, CVD related “indirect” costs from reduced employment, premature death, absenteeism, and presenteeism were estimated at AUD 6.2 billion in 2015–16 figures [12,13]. In the same year, the health expenditure from endocrine disorders, including Type 2 Diabetes Mellitus (T2DM), was estimated at AUD 3.5 billion [11].

Diets low in whole grain are ranked as the second leading issue in terms of the global burden of disease and, together with the current less than ideal level of whole grain consumption, there is impetus to examine the potential of dietary change, particularly in order to reduce healthcare expenditure. A previous cost-of-illness-analysis on cereal fibre has been conducted using Australian data [12]; however, the dietary fibre of whole grains only partly explains their beneficial health effects [14]. Barret et al. [15] recently found that whole grain and cereal fibre intake were both independently associated with protection against various CVD risk factors. For instance, whereas whole grain intake was associated with desirable blood glucose measures, cereal fibre intake was associated with desirable blood lipid measures, supporting the suggestion that components of the whole grain, such as dietary fibre, magnesium, potassium, selenium, zinc, iron, iodine, folate, niacin, vitamin E, and phytochemicals work synergistically in producing such health effects. The objective of this study was thus to estimate the potential savings in costs of healthcare and lost productivity that would be associated with reductions in the incidences of T2DM and CVD, separately, following the DTI for whole grains across the Australian population.

## 2. Materials and Methods

### 2.1. Financial Model Design

Building on the relevant scientific literature and monetary figures from national databases, a three-step cost-of-illness analysis was developed to assess the potential short- and long-term savings in costs of healthcare and productivity loss by using input parameters from: (1) estimates of current and targeted whole grain intakes among proportions of the Australian adult population; (2) estimates of reductions in relative risk of T2DM and CVD that are associated with specific intakes of whole grains from meta-analysis studies, with corresponding lower and upper 95% confidence intervals; and (3) estimates of annual healthcare (direct) and lost productivity (indirect) costs associated with T2DM and CVD in Australia. Similar to previous research [13], a sensitivity analysis of very pessimistic, pessimistic, optimistic, and universal scenarios was applied to parameters of the three steps of the analysis, corresponding to the whole grain uptake rate, impact on the two diseases of interest, and the healthcare cost flexibility. Input parameters of the economic modelling are summarised in Table 1, and further details are provided below.

### 2.2. Step 1: Uptake Rate (Estimates of Proportions of Prospective Consumers)

The first step of this analysis was based on dietary intake data from the 2011–2012 NNPAS (*n* = 12,153) [6]. Single 24 h recalls were administered using the Automated Multiple-Pass Method by trained interviewers. Details of dietary interview methods, including the Food Model Booklet used by interviewers in describing amounts of food and beverages consumed, are provided elsewhere [20]. A secondary analysis of the survey [6] estimated current median daily whole grain intake at 21 g for adults (19–85 years), and this was compared to an established DTI of 48 g [9,16,17]. The calculation was then built on estimates of proportions of Australian adults (20 y and over) who are likely to increase their intake of whole grains by the “gap” amount of 27 g daily. In applying a sensitivity analysis in this step, the very pessimistic and pessimistic scenarios, respectively, represent the practical short-term and short-to-medium-term estimates of cost savings that could follow when 5% and 15% of Australian adults increase their intake of whole grains to the DTI of 48 g. The optimistic scenario is a medium-to-long-term pragmatic estimate of potential savings when 50% of adults adopt the target intake of whole grains. Additionally, the universal scenario is a best-case long-term estimate of potential savings, when 100% of adults increase their intake of whole grains. It is important to note that 73% of all Australians >9 years of age consumed less than the 48 g DTI, and 30% were considered non-consumers of whole grain [6].

### 2.3. Step 2: Disease Risk Reduction (Estimates of Reductions in Relative Risk of T2DM and CVD)

In assessing the percent reductions in T2DM and CVD incidences that would potentially correspond to the mean gap of consumption between the current intake and DTI of whole grains in Australia (Step 1), and upon a key word search of the relevant literature, the second step of this analysis employed data from two systematic reviews and dose–response meta-analyses of prospective cohort studies by Aune et al. [18,19], respectively. The first of these meta-analyses included 16 studies (10 reporting on whole grain intake and T2DM risk among 385,868 participants) and suggested a relative risk (RR) per 3 servings (using 30 g of food as a serving size) per day, equal to 0.68 (95% CI 0.58–0.81) for whole grains and 0.95 (95% CI 0.88–1.04) for refined grains [18]. The second meta-analysis included 45 studies (10 reporting on whole grain intake and CVD risk among 704,317 participants) and suggested an RR per 90 g/day (equivalent to 3 servings) of 0.87 (95% CI 0.78–0.97) for CVD incidence and 0.78 (95% CI 0.73–0.85) for incidence or mortality [19]. Building on these RRs while assuming a linear relationship, an RR reduction per 27 g whole grain intake was calculated (as shown in Table 1) for use in the final step of the analysis. At the time when the Aune et al. [18,19] studies were published, there was no global definition of whole grain, and the weight of products was used to determine a serving of whole grains (e.g., one 30 g slice of wholemeal bread). In this analysis, 30 g of whole grain “product” was assumed to be equivalent to 16 g of whole grain “content”, with three servings being in line with the recommended DTI of 48 g. This conversion was recommended by the recent work of Ross et al. [21] and utilised in another analysis in the US by Murphy and Schmier [22].

### 2.4. Step 3: Disease Cost Savings (Estimates of Annual Savings in Healthcare and Lost Productivity Costs)

In the third and final step of this analysis, annual savings in costs of healthcare and lost productivity that could follow the estimated reductions in incidences of T2DM and CVD (Step 2) with the recommended increase in consumption of whole grains were calculated. Disease expenditure data from the AIHW (2015–2016) [11] and others [23,24] were employed, with an adjustment of inflation rates according to the Australian Bureau of Statistics (ABS) Consumer Price Index (Health group) [25] (Table 2). Costs of disease are typically broken down into direct and indirect measures, where the former refers to healthcare-related costs of hospital admission and services, prescriptions, and general practitioner services, whereas the latter refers to productivity- and mortality-related costs. Similar to previous analyses in a Canadian population [13,26], the savings figures were estimated for each component of the direct healthcare and indirect productivity loss expenditures individually, based on the assumption that each 1% decline in disease incidence would translate to a 1% reduction in cost of that component.

### 2.5. Discounted Rate

The discount rate is defined as the interest rate that converts future monetary figures into present values [28]. Following the methodology outlined in detail previously [26], a real discount rate of 7% was applied to the total savings in T2DM and CVD cost data, separately, to assess the discounted value of whole grain consumption over the longer term, using the net present value equation:

(1) $$Discounted\;cost\;savings=savings\;at\;year\;0\;x\;\frac{1}{{\left(1+r\right)}^{n}}$$

where *r* = real discount rate (7%) and *n* = years into the future. The equation and conservative discount rate of 7% have been used across almost all Australian jurisdictions [29,30,31,32]. The real discount rate was applied to present day savings in T2DM and CVD healthcare costs at five-year increments for 20 years after 2020 (year 0), i.e., 0–4 years (very pessimistic), 5–9 years (pessimistic), 10–14 years (optimistic), and 15–19 years (universal).

## 3. Results

Using input parameters from the three steps of the current analysis (Table 1), the total annual savings in direct healthcare and productivity loss expenditures associated with the reductions in incidence of T2DM and CVD following the 48 g DTI against the 21 g/day current median intake of whole grains across the Australian adult population were estimated at AUD 37.5 (95% CI 22.3–49.3) to AUD 750.7 (95% CI 445.7–985.2) million (Table 3) and AUD35.9 (95% CI 8.3–60.7) to AUD 717.4 (95% CI 165.5–1214.1) million (Table 4), respectively.

To account for the longer timeframe required to achieve success, using the discount rate of 7% as per the Australian Governments recommendations [29], the sum of savings in healthcare and productivity loss expenditure associated with the reductions in incidences of the two diseases of interest following the 48 g DTI of whole grains was estimated at AUD 2547.6 (95% CI 1512.6–3343.7) million and AUD 2434.8 (95% CI 561.9–4120.4) million for T2DM and CVD, respectively (Table 5). This allows for increasing the success over a 20-year period in adoption of whole grains and, in line with this, substantially greater cost savings would accrue, particularly if T2DM and CVD are considered together.

## 4. Discussion

This cost-of-illness analysis supports a greater focus on whole grains as part of the current dietary pattern, demonstrating a potentially substantial saving for the Australian healthcare system in the costs for both T2DM and CVD. Grain foods are already central to the Australian diet, although a large proportion is consumed as refined grain, white bread, white rice, white pasta, and white flour products such as cakes, scones, and biscuits. Exchanging whole grains for refined grains to levels that meet the DTI among 5% up to 100% of Australian adults is estimated to lead to between AUD 37.5 and 750.7 million and AUD 35.9 to 717.4 million in total annual healthcare- and productivity-related costs for T2DM and CVD, respectively. A similar analysis was conducted in the US, where estimated healthcare savings related to CVD from modelled increases of 0.25 oz-eq per day (~4 g) of whole grains was USD 2.4 billion (USD 0.6 to USD 4.3) [22]. The larger valuation in the US compared to the results in this study is likely due to the larger population size and higher cardiovascular related healthcare costs.

The DTI for whole grains in Australia is suggested as 48 g, or three servings of whole grain foods (16 g/serving) [9,33]. Australia’s DTI for whole grains and corresponding serving sizes are aligned to recommendations in the literature [16,34]. Three servings can be easily achieved by exchanging food items rather than adding to the energy density of the diet. For example, by targeting breakfast cereals and wholemeal bread, the two largest sources of whole grain within the current Australian diet [6], target levels for whole grains could be achieved with a minimal burden on consumers. A recent analysis of the breakfast cereal category suggests 67% of products (*n* = 364) are considered whole grain, and these have an improved nutritional profile compared to products that are refined or below the cut-off for whole grain content (<8 g/manufacturer serving) in terms of sodium and sugars [35]. A similar analysis of the Australian bread market found that one-third of breads on supermarket shelves were whole grain/wholemeal, with a median whole grain content of 20.2 g per serving (2 slices), almost half of the 48 g DTI [36].

The health benefits of whole grain products are well established. With the exception of Denmark, where 100% whole grain rye bread is frequently consumed, consumption remains relatively low globally. In the last NNPAS (2011–12), Australians consumed, on average, 4.5 servings of grain (cereal) foods from non-discretionary sources per day, with men consuming more than women (5.1 versus 3.8 servings) [2]. Dietary guidelines recommend that two-thirds of grains should be whole grain (or high dietary fibre varieties), whereas only around one-third (34%) were from whole grain or high fibre sources. A re-analysis of the NNPAS data indicated that the median daily intake of whole grains was 21 g and 17 g for adults (19–85 years) and children/adolescents (2–18 years), respectively [6]. This analysis found that 30% of children/adolescents consumed no whole grain on the day of the survey, and average intake of whole grains among adolescents was 8.7 g/d [6]. Older Australians performed best, with those over 71 years consuming 55% of their grains as whole grains [6,37]. Based on our data, if consumption of the DTI for whole grains could be increased by 15% to 50% in the adult population, the annual savings would reach AUD 112.6 to 375.3 million and AUD 107.6 to 358.7 million in costs of T2DM and CVD, respectively.

A similar cost-of-illness analysis demonstrated an increasing consumption in cereal fibre by Australians could facilitate annual healthcare and lost productivity cost-savings of AUD 18.2 million to 1.7 billion for T2DM and AUD 17.8 million to 1.6 billion for CVD [12]. The healthcare and productively savings with cereal fibre were substantially greater than results from the present study. Interestingly, as we have noted, dietary fibre from grains (cereal foods) underscores the total benefit of consuming the whole grain. Additionally, in the cost-of-illness analysis on cereal fibre, the authors suggested consuming whole grain foods such as wholemeal bread to reach the Suggested Dietary Target (SDT) for the prevention of chronic disease. This may entail adding high cereal fibre foods to the diet, increasing grain foods servings and therefore impacting the energy density of the diet. In swapping to whole grain, both dietary fibre and whole grain goals can be achieved without necessarily increasing energy intake.

Since the last NNPAS, there has been growth in the number of whole grain products on the Australian market, supported by the Grains and Legumes Nutrition Council (GLNC) Code of Practice for Whole Grain Ingredient Content Claims (The Code) [38]. From the inception of The Code in mid-2013 up until mid-2019, there was a 71% increase in the number of products making whole grain claims [39] and, in almost two years since then, there are now more than 1000 whole grain products registered with The Code in Australia and New Zealand. This is a similar number as in Denmark, although product numbers alone do not facilitate greater consumption. The Code provides the necessary framework for industry and outlines a minimum whole grain content of 8 g per manufactured serving to make the claim, which is based on the Australian Dietary Guidelines’ minimum suggestion of six servings of grain foods each day [5], combined with the 48 g DTI. Permitted content claims are based on three levels of whole grains ≥8 g (“contains whole grains”), ≥16 g (“high in whole grains”), and ≥24 g per manufacturer serving (“very high in whole grains”). Claims can also be factual (such as “100% whole grain” or “22 g whole grains per serving”) or related to the percentage contribution towards the 48g DTI on a per serving basis, known as a “DTI statement” [38]. This provides a level of clarity and transparency across all whole grain foods and is monitored by GLNC.

Many countries actively promote whole grains, including the US [40], UK [41], Canada [42], Denmark [43], France [44], Germany [45], and Singapore [46], and there are public–private partnerships established in the US, Denmark, and Australia and organizations based in Europe, such as the Health Grain Forum and the Whole Grain Initiative; the latter has the goal of uniting organisations and industry to improve the food supply. Despite the significant body of research supporting whole grains, and the global burden of disease data, the suggestion to consume whole grains is often not the focus of the grain food statement in dietary guidelines. Some guidelines fail to mention whole grains and, frequently, passive words such as “preferably” or “mostly” are used to promote the consumption of whole grains [3]. In contrast, in Denmark [43], where greater increases in whole grain have been achieved in recent years, the recommendation is to simply “Choose Whole Grain” and, in Canada, the most recent visual guide only depicts whole grain food options [42].

Whole grain content is also underutilized in front-of-pack labelling initiatives, an important step in consumer recognition and a factor that may be able to help differentiate refined and whole grain products on the supermarket shelf. Research has demonstrated that the health star rating system is inadequate in highlighting whole grain food choices to consumers, with less than a star separating the higher quality whole grain products from refined varieties across several food categories including bread, rice, noodles, flour, breakfast cereal, and muesli bars [47]. Others have raised similar concerns regarding the Nutri-score and Nutrient Rich Food Index [48]. Currently there are six front-of-pack tools incorporating whole grains—the Singapore Healthier Choices logo, the Keyhole (in Sweden, Denmark and Norway), the Weqaya logo (in the United Arab Emirates), the Healthy Living Guarantee (Croatia), the Healthier Choice Logo (in Malaysia), and the Healthier Choice Symbol (in Brunei) [49], proving that the addition of whole grain content to the algorithm used in the various scoring tools is not impossible.

As in other parts of the world, Australians, in general, are non-compliant with many of the suggested dietary targets included in dietary guidelines [50]. Falling short on protective foods high in dietary fibre, such as fruit, vegetables, legumes, nuts, and seeds, the adequacy of whole grain foods is just one of many aspects to be considered in policy. Additionally, there are impacts from less desirable food and drink choices on health outcomes. Other cost–benefit studies based on the Australian population have included dairy products, suggesting increased consumption to 2–3 servings per day and yielding AUD 1.1–3.8 billion [51]; another examined the impact of discretionary foods on obesity with a reduction in one serving of a sugar-sweetened beverage, yielding AUD 793.4 million in healthcare cost-savings [52]. Another study assessed two potential methods for reducing alcohol (through pricing or taxation) and its impact on obesity, yielding AUD 3.3–5.8 billion. Smaller cost reductions have also been calculated for reducing trans fats in relation to ischemic heart disease, with savings of AUD 7.2–212.9 million, noting that intake, on average, is already low, and levels have not changed substantially in the last ten years [53]. There are limitations to considering single dietary components. As such, other ecconomic modelling studies considered multiple diet and lifestyle changes [54,55]. A Canadian study focused on the food categories important in the global burden of disease, where both protective foods (fruit, vegetables, milk, whole grains, nuts, and seeds) and “harmful foods” (sugar-sweetened beverages, processed meat, and red meat) realised CAD 15.8 billion/year, with a greater economic burden coming from under-consuming healthy foods (CAD 12.5 billion) versus the overconsumption of harmful foods (CAD 3.3 billion) [50]. This suggests that a greater focus on healthy foods such as whole grains, fruit, vegetables, legumes, and nuts is likely to result in more effective policy and greater cost-savings than targeting less desirable food groups, a concept also suggested in the global burden of disease studies [7].

A potential limitation to the 100% adoption scenario across the Australian population may be due to issues with gluten among those suffering with coeliac disease or other related conditions (1–2% of the population). There are, however, a number of gluten-free grains or pseudo grains, including quinoa, buckwheat, and amaranth, which may be used in a gluten-free diet prescription. The avoidance of wheat, gluten, or grains may also include those who are intolerant to wheat (non-coeliac wheat sensitivity), including those following a low FODMAP diet (fermentable oligo-, di-, monosaccharides and polyols), although this is usually a temporary dietary pattern [56]. It has been estimated that an additional 7% of Australians (3.1 million people) avoid gluten and/or wheat in their diets [57].

A particular strength of the current study are the calculations relating to the dicounted rate, which acknowledges that costs inccurred have a greater value in the current day, and improvements in the dietary pattern from increasing whole grain intake would provide an immediate benefit in healthcare cost savings [28]. However, as dietary adoptions and behaviour changes would be gradual across the whole population, an adjustment for time is required, signalling that, although supportive measures need to be ongoing to realise results, achieving this in the shortest timeframe possible is most beneficial. The most recent data suggest that 27% of the population meets the 48 g DTI; however, the savings at 50% adoption would realise several million dollars in savings more than the current level if this were achieved within a 20-year timeframe, and a doubling of that again if closer to 100% could be achieved.

Future models using cost-saving analyses should perhaps consider age more closely, and the added benefits of correcting diet earlier in life, as the beneficial effects of whole grains are more likely the result of longer-term dietary patterns and are, therefore, accompanied by greater cost-savings over time. A significantly larger proportion of those aged >51 years met the 48 g target (42.4%) in the NNPAS compared to the 19–50 years age group (38.4%), while adolescents (14–18 years, both males and females) had far lower intakes (8.7 g/day) [6]. As expected, there are suggestions of a greater benefit in correcting whole grain intake in groups with the lowest consumption [16]. Non-consumers made up 28.8% of the adult population and 29.9% of children, so these are also an appropriate target, but they may be more difficult to influence than the low-level consumers. The current cost-saving analyses did not consider the differences in whole grain consumption between males and females, which may also be an area for future research. Adjusting for socioeconomic status would also be relevant, as savings were larger among adults of lower socioecconomic status in the study of cereal fibre [12]. With this in mind, the cost of whole grain food items may also be a barrier, except that most products are at price parity to refined varieties, with the exception being bread, which has been identified in Canadian research [58]. In the context of the whole diet, grain and cereal foods are less expensive relative to other categories, delivering a range of essential nutrients and at risk micronutrients [59,60]. Furthermore, a study examining the typical Australian diet basket with the EAT Lancet Commissions Planetary Health Diet found savings for families in selecting the suggested dietary pattern from EAT Lancet (AUD 224.4 versus AUD 188.2), with double the grams in weight of whole grains, allowing all family members to reach the 48 g DTI.

## 5. Conclusions

There are substantial healthcare cost-savings, greater than AUD 1.4 billion, for T2DM and CVD, with an increasing proportion of Australians meeting the 48 g whole grain DTI. The emphasis in current promotion should be focused on swapping refined grain choices for whole grains. Energy balance is an important consideration in public health advice, as more than half of Australians are overweight or obese; this change in food choice would unlikely impact the energy density of the diet. As has been suggested, the focus on protective foods in policy and food-based dietary guidelines, such as whole grains, may yield more favourable cost-saving results for future healthcare than a focus on foods to avoid. Research has identified a potential reduction in disease with each additional serving of whole grains, up to 48 g/day, which is an important message for heathcare providers and policy makers.

## Figures and Tables

**Table 1:** Summary of the cost-of-illness analysis input parameters and corresponding references.

Parameter	Men and Women	Reference
Current whole grain intake, g/day	21	Galea et al. [6]
Daily Target Intake, g	48	Griffiths et al., Chen et al., Zong et al. [9,16,17]
Mean gap in consumption, g/day	27	
Uptake rate (proportions of prospective consumers) ^1^	Very pessimistic (5%), pessimistic (15%), optimistic (50%), universal (100%)	Estimates
T2DM incidence		
Relative risk reduction per 90 g whole grain/day (95% CI)	−32% (19–42)	Aune et al. [18]
Relative risk reduction per 27 g whole grain/day (95% CI) ^2^	−9.6% (5.7–12.6)	Calculation
CVD incidence		
Relative risk reduction per 90 g whole grain/day (95% CI)	−13% (3–22)	Aune et al. [19]
Relative risk reduction per 27 g whole grain/day (95% CI) ^2^	−3.9% (0.9–6.6)	Calculation

**Abbreviations:** CVD, cardiovascular disease; T2DM, Type 2 Diabetes Melllitus. ^1^ Estimates of proportions of the Australian adult population (20 y and over) who would increase their whole grain consumption to the recommended DTI level of 48 g over the short term (very pessimistic), short-to-medium term (pessimistic), medium-to-long term (optimistic), and long term (universal). ^2^ Risk reduction per 27 g was calculated from the reported values by Aune et al. [18] or [19] of 90 g/day, assuming a linear relationship.

**Table 2:** Summary of Type 2 Diabetes Mellitus and cardiovascular disease direct health and productivity loss expenditures in Australia (AUD million), age 20 and over.

	T2DM		CVD	
	2015–2016	2020 ^2^	2015–2016	2020 ^2^
Direct health expenditure ^1^				
Allied health and other services	29.8	33.9	19.4	22.1
General practitioner services	109.0	124.0	714.4	813.2
Medical imaging	1.5	1.7	147.6	168.0
Pathology	78.0	88.8	188.6	214.7
Pharmaceutical benefits scheme ^3^	311.9	355.0	1650.1	1878.3
Private hospital services	64.5	73.5	2268.2	2581.8
Public hospital admitted patient	435.6	495.8	3668.0	4175.2
Public hospital emergency department ^4^	2.2	2.5	485.3	552.4
Public hospital outpatient	130.6	148.6	430.4	489.9
Specialist services	25.7	29.2	376.5	428.5
All direct health expenditure	1188.6	1353.0	9948.5	11,324.0
Productivity loss expenditure ^5^				
Reduced employment	1443.7	1643.3	3248.9	3698.1
Premature death	279.9	318.6	1937.4	2205.3
Absenteeism	324.3	369.1	141.9	161.5
Presenteeism	3633.1	4135.4	884.2 ^6^	1006.5
All productivity loss expenditure	5681.0	6466.5	6212.4	7071.3
**Total expenditure**	**6869.6**	**7819.4**	**16,160.9**	**18,395.4**

**Abbreviations:** AUD, Australian dollar; CVD, cardiovascular disease; T2DM, Type 2 Diabetes Mellitus. ^1^ From the Australian Institute of Health and Welfare disease expenditure database (2015–2016) [11]. ^2^ Current AUD based on adjustment of inflation rates according to the Australian Bureau of Statistics Consumer Price Index (Health group) [25]. ^3^ Includes over and under copayment prescriptions. ^4^ Exclude the ACT for this data period. ^5^ From Fayet-Moore et al. [12] based on data for type I and II diabetes [27] and data for cardiovascular disease [23] ^6^ From Fayet-Moore et al. [12] based on presenteeism costs for stroke [24].

**Table 3:** Potential annual savings in direct health and productivity loss expenditures of Type 2 Diabetes Mellitus in Australian adults (age 20 and over) from whole grain intakes (AUD million) ^1^.

	Scenario			
	Very Pessimistic	Pessimistic	Optimistic	Universal
Direct health expenditure savings				
Allied health and other services	0.2 (0.1–0.2)	0.5 (0.3–0.6)	1.6 (1.0–2.1)	3.3 (1.9–4.3)
General practitioner services	0.6 (0.4–0.8)	1.8 (1.1–2.3)	6.0 (3.5–7.8)	11.9 (7.1–15.6)
Medical imaging	<0.01 (<0.01–<0.01)	<0.01 (<0.01–<0.01)	0.1 (<0.01–0.1)	0.2 (0.1–0.2)
Pathology	0.4 (0.3–0.6)	1.3 (0.8–1.7)	4.3 (2.5–5.6)	8.5 (5.1–11.2)
Pharmaceutical benefits scheme	1.7 (1.0–2.2)	5.1 (3.0–6.7)	17.0 (10.1–22.4)	34.1 (20.2–44.7)
Private hospital services	0.4 (0.2–0.5)	1.1 (0.6–1.4)	3.5 (2.1–4.6)	7.1 (4.2–9.3)
Public hospital admitted patient	2.4 (1.4–3.1)	7.1 (4.2–9.4)	23.8 (14.1–31.2)	47.6 (28.3–62.5)
Public hospital emergency department	<0.01 (<0.01–<0.01)	<0.01 (<0.01–<0.01)	0.1 (0.1–0.2)	0.2 (0.1–0.3)
Public hospital outpatient	0.7 (0.4–0.9)	2.1 (1.3–2.8)	7.1 (4.2–9.4)	14.3 (8.5–18.7)
Specialist services	0.1 (0.1–0.2)	0.4 (0.2–0.6)	1.4 (0.8–1.8)	2.8 (1.7–3.7)
All direct health savings	6.5 (3.9–8.5)	19.5 (11.6–25.6)	64.9 (38.6–85.2)	129.9 (77.1–170.5)
Productivity loss expenditure savings				
Reduced employment	7.9 (4.7–10.4)	23.7 (14.1–31.1)	78.9 (46.8–103.5)	157.8 (93.7–207.1)
Premature death	1.5 (0.9–2.0)	4.6 (2.7–6.0)	15.3 (9.1–20.1)	30.6 (18.2–40.1)
Absenteeism	1.8 (1.1–2.3)	5.3 (3.2–7.0)	17.7 (10.5–23.3)	35.4 (21.0–46.5)
Presenteeism	19.9 (11.8–26.1)	59.6 (35.4–78.2)	198.5 (117.9–260.5)	397.0 (235.7–521.1)
All productivity savings	31.0 (18.4–40.7)	93.1 (55.3–122.2)	310.4 (184.3–407.4)	620.8 (368.6–814.8)
**Total savings**	**37.5 ** **(22.3–49.3)**	**112.6 ** **(66.9–147.8)**	**375.3 ** **(222.9–492.6)**	**750.7 ** **(445.7–985.2)**

**Abbreviations:** AUD, Australian dollar. ^1^ Data (95% CI) are monetary savings following Type 2 Diabetes Mellitus incidence risk reduction with whole grain intake (Table 1). The very pessimistic and pessimistic scenarios are, respectively, practical short-term and short-to-medium-term estimates of expenditure savings that could follow when 5% and 15% of Australian adults (age 20 and over) consume the daily target intake of whole grains. The optimistic scenario is a medium-to-long-term pragmatic estimate of potential savings when 50% of adults in Australia adopt the recommended level of whole grains. The universal scenario is a best-case long-term estimate of potential savings when 100% of Australian adults increase their intake of whole grains.

**Table 4:** Potential annual savings in direct health and productivity loss expenditure of cardiovascular disease in Australian adults (age 20 and over) from whole grain intakes (AUD million) ^1^.

	Scenario			
	Very Pessimistic	Pessimistic	Optimistic	Universal
Direct health expenditure savings				
Allied health and other services	<0.01 (<0.01–0.1)	0.1 (<0.01–0.2)	0.4 (0.1–0.7)	0.9 (0.2–1.5)
General practitioner services	1.6 (0.4–2.7)	4.8 (1.1–8.1)	15.9 (3.7–26.8)	31.7 (7.3–53.7)
Medical imaging	0.3 (0.1–0.6)	1.0 (0.2–1.7)	3.3 (0.8–5.5)	6.6 (1.5–11.1)
Pathology	0.4 (0.1–0.7)	1.3 (0.3–2.1)	4.2 (1.0–7.1)	8.4 (1.9–14.2)
Pharmaceutical benefits scheme	3.7 (0.8–6.2)	11.0 (2.5–18.6)	36.6 (8.5–62.0)	73.3 (16.9–124.0)
Private hospital services	5.0 (1.2–8.5)	15.1 (3.5–25.6)	50.3 (11.6–85.2)	100.7 (23.2–170.4)
Public hospital admitted patient	8.1 (1.9–13.8)	24.4 (5.6–41.3)	81.4 (18.8–137.8)	162.8 (37.6–275.6)
Public hospital emergency department	1.1 (0.2–1.8)	3.2 (0.7–5.5)	10.8 (2.5–18.2)	21.5 (5.0–36.5)
Public hospital outpatient	1.0 (0.2–1.6)	2.9 (0.7–4.9)	9.6 (2.2–16.2)	19.1 (4.4–32.3)
Specialist services	0.8 (0.2–1.4)	2.5 (0.6–4.2)	8.4 (1.9–14.1)	16.7 (3.9–28.3)
All direct health savings	22.1 (5.1–37.4)	66.2 (15.3–112.1)	220.8 (51.0–373.7)	441.6 (101.9–747.4)
Productivity loss expenditure savings				
Reduced employment	7.2 (1.7–12.2)	21.6 (5.0–36.6)	72.1 (16.6–122.0)	144.2 (33.3–244.1)
Premature death	4.3 (1.0–7.3)	12.9 (3.0–21.8)	43.0 (9.9–72.8)	86.0 (19.8–145.5)
Absenteeism	0.3 (0.1–0.5)	0.9 (0.2–1.6)	3.1 (0.7–5.3)	6.3 (1.5 −10.7)
Presenteeism	2.0 (0.5–3.3)	5.9 (1.4–10.0)	19.6 (4.5–33.2)	39.3 (9.1–66.4)
All productivity savings	13.8 (3.2–23.3)	41.4 (9.5–70.0)	137.9 (31.8–233.4)	275.8 (63.6–466.7)
**Total savings**	**35.9 (8.3–60.7)**	**107.6 (24.8–182.1)**	**358.7 (82.8–607.0)**	**717.4 (165.6–1214.1)**

**Abbreviations:** AUD, Australian dollar. ^1^ Data (95% CI) are monetary savings following cardiovascular disease incidence risk reduction with whole grain intake (Table 1). The very pessimistic and pessimistic scenarios are, respectively, practical short-term and short-to-medium-term estimates of expenditure savings that could follow when 5% and 15% of Australian adults (age 20 and over) consume the daily target intake of whole grains. The optimistic scenario is a medium-to-long-term pragmatic estimate of potential savings when 50% of adults in Australia adopt the recommended level of whole grains. The universal scenario is a best-case long-term estimate of potential savings when 100% of Australian adults increase their intake of whole grains.

**Table 5:** Sum of potential total discounted savings on direct health and productivity loss expenditures of Type 2 Diabetes Mellitus and cardiovascular disease in Australian adults (age 20 and over) from whole grain intakes over short-term and long-term periods (AUD million) ^1^.

	Scenario			
	Very Pessimistic	Pessimistic	Optimistic	Universal
*Total savings of years 0 to 4*				
T2DM	**164.7 (97.8–216.1)**	494.0 (293.3–648.4)	1646.7 (977.7–2161.2)	3293.3 (1955.4–4322.5)
CVD	**157.4 (36.3–266.3)**	472.1 (109.0–799.0)	1573.7 (363.2–2663.2)	3147.5 (726.3–5326.5)
*Total savings of years 5 to 9*				
T2DM	117.4 (69.7–154.1)	**352.2 (209.1–462.3)**	1174.0 (697.1–1540.9)	2348.1 (1394.2–3081.9)
CVD	112.2 (25.9–189.9)	**336.6 (77.7–569.7)**	1122.1 (258.9–1898.9)	2244.1 (517.9–3797.7)
*Total savings of years 10 to 14*				
T2DM	83.7 (49.7–109.9)	251.1 (149.1–329.6)	**837.1 (497.0–1098.7)**	1674.2 (994.0–2197.3)
CVD	80.0 (18.5–135.4)	240.0 (55.4–406.2)	**800.0 (184.6–1353.9)**	1600.0 (369.2–2707.7)
*Total savings of years 15 to 19*				
T2DM	59.7 (35.4–78.3)	179.0 (106.3–235.0)	596.8 (354.4–783.3)	**1193.7 (708.7–1566.7)**
CVD	57.0 (13.2–96.5)	171.1 (39.5–289.6)	570.4 (131.6–965.3)	**1140.8 (263.3–1930.6)**
**Total discounted savings for each scenario (2020–2039)**				
**T2DM**	**425.5 (252.6–558.4)**	**1276.4 (757.9–1675.3)**	**4254.6 (2526.2–5584.2)**	**8509.2 (5052.4–11,168.4)**
**CVD**	**406.6 (93.8–688.1)**	**1219.9 (281.5–2064.4)**	**4066.2 (938.4–6881.2)**	**8132.4 (1876.7–13,762.5)**
**Total incremental discounted savings: adoption of each scenario every 5 years (2020–2039)**				
**T2DM**	**-**	**-**	**-**	**2547.6 (1512.6–3343.7)**
**CVD**	**-**	**-**	**-**	**2434.8 (561.9–4120.4)**

**Abbreviations:** AUD, Australian dollar; CVD, cardiovascular disease; T2DM, Type 2 Diabetes Mellitus. ^1^ Data (95% CI) are monetary savings following Type 2 Diabetes Mellitus and cardiovascular disease incidence risk reductions with whole grain intake. The very pessimistic and pessimistic scenarios are, respectively, practical short-term and short-to-medium-term estimates of expenditure savings that could follow when 5% and 15% of Australian adults (age 20 and over) consume the daily target level of whole grains. The optimistic scenario is a medium-to-long-term pragmatic estimate of potential savings when 50% of adults in Australia adopt the recommended level of whole grains. The universal scenario is a best-case long-term estimate of potential savings when 100% of Australian adults increase their intake of whole grains.

## Data Availability

All data for this study is contained within the article.
